# Costs of illness of multiple sclerosis in Sweden: a population-based register study of people of working age

**DOI:** 10.1007/s10198-017-0894-6

**Published:** 2017-05-09

**Authors:** Hanna Gyllensten, Michael Wiberg, Kristina Alexanderson, Anders Norlund, Emilie Friberg, Jan Hillert, Olivia Ernstsson, Petter Tinghög

**Affiliations:** 1 Department of Clinical Neuroscience, Karolinska Institutet, Berzelius Väg 3, Floor 6, SE-171 77 Stockholm, Sweden; 2 Institute of Health and Care Sciences, Sahlgrenska Academy, University of Gothenburg, Box 457, SE-405 30 Gothenburg, Sweden; 3 Department of analysis and prognosis, Swedish Social Insurance Agency, SE-126 37 Stockholm, Sweden; 4 Department of Learning, Informatics, Management and Ethics, Karolinska Institutet, SE-171 77 Stockholm, Sweden; 5 Red Cross University College, Teknikringen 1, SE-114 28 Stockholm, Sweden

**Keywords:** Multiple sclerosis, Cost of Illness, Registries, Third-party payers, Socioeconomic factors, Sick leave, I140, I180, H510, H550

## Abstract

**Background:**

Multiple sclerosis (MS) causes work disability and healthcare resource use, but little is known about the distribution of the associated costs to society.

**Objectives:**

We estimated the cost of illness (COI) of working-aged individuals with MS, from the societal perspective, overall and in different groups.

**Methods:**

A population-based study was conducted, using data linked from several nationwide registers, on 14,077 individuals with MS, aged 20–64 years and living in Sweden. Prevalence-based direct and indirect costs in 2010 were calculated, including costs for prescription drug use, specialized healthcare, sick leave, and disability pension.

**Results:**

The estimated COI of all the MS patients were SEK 3950 million, of which 75% were indirect costs. MS was the main diagnosis for resource use, causing 38% of healthcare costs and 67% of indirect costs. The distribution of costs was skewed, in which less than 25% of the patients accounted for half the total COI.

**Conclusions:**

Indirect costs contributed to approximately 75% of the estimated overall COI of MS patients of working age in Sweden. MS was the main diagnosis for more than half of the estimated COI in this patient group. Further studies are needed to gain knowledge on development of costs over time during the MS disease course.

## Introduction

Multiple sclerosis (MS) is a neurological, often progressive disease and the most common degenerative neurological disease in people of working age [1]. Most individuals are diagnosed when aged 20–40 years [2] and the disease often results in different types of disability that impact work participation and cause sickness absence and disability pension. Thus, MS leads to both work disability and healthcare resource use. It has been estimated that, in 2005, the annual cost of MS in Sweden was 600 million euros [3]. However, previous estimations of the economic impact of MS in society have predominantly been limited to information from questionnaires distributed to patients either attending a specific healthcare unit or who were members of a specific patient organization, and response rates have sometimes been low (and often not reported); the range was 16–99%, [4]. Thus, little is known about the representativeness of the estimated impact from those studies for all individuals with MS (hereafter called MS patients) in a country [4]. Regarding a disease like MS, with large recent and ongoing changes in the treatment, up-to-date knowledge of costs is necessary for real-world cost-effectiveness assessment when implementing and making decisions on reimbursement of new treatments [5], but such estimates also need to be representative of costs in the whole patient population.

Studies of the economic burden of diseases are often referred to as cost-of-illness (COI) studies; such studies are used for estimating the economic impact of a disease in society, and the distribution of costs between payers [6–8]. In most countries, costs affect different authorities or organizations. In Sweden, for example, county councils are responsible for organizing healthcare while another authority handles the bulk part of sick leave and disability pension benefits, namely the Social Insurance Agency [9]. To ensure that a wide spectrum of economic consequences in society are accounted for, the COI can be measured from a societal perspective, including both direct (opportunity costs of resources used, such as healthcare and drug use) and indirect costs (productivity costs: costs resulting from lost productivity due to, e.g., morbidity) [10].

In addition, several sociodemographic factors may affect the development of MS [2] or the COI of MS patients. Comorbidities, such as mental disorders, are common among MS patients [11], and the occurrence of comorbidities has been associated with higher disability pension prevalence among MS patients [12]. Thus, different attempts to elucidate aspects of comorbidities for cost outcomes are warranted.

The aim of this study was to estimate the societal COI of MS patients of working age in Sweden and to specify the distribution of those costs. Another aim was to explore the distribution of costs between diagnoses (both MS and other diagnosis groups) among MS patients. Finally, to investigate if the costs resulting from resource use with MS as the main diagnosis varied by sex, socioeconomic factors, and years since being diagnosed with MS.

## Materials and methods

### Participants and data collection

In this cross-sectional nationwide population-based study, the study population consisted of all individuals previously diagnosed with MS who were aged 20–64 years on 31 December 2010 and lived in Sweden all of 2010. Data from several nationwide registers was used, linked by the personal identification number each resident in Sweden is given.

Individuals with MS were identified based on diagnosis information from the National Patient Register (PAR), from the National Board of Health and Welfare, and from sick leave and disability pension diagnoses from the Micro-Data for Analysis of the Social Insurance System (MiDAS) register at the Social Insurance Agency [13]. Individuals with at least one International Classification of Disease (ICD), versions 9 or 10, code indicating MS (340 and G35, respectively) were included as MS patients, including all diagnoses available in the registers up to and including the year 2010. From PAR, information on MS diagnoses from inpatient care was identified from 1987 onwards, and from specialized outpatient care from 2001. Disability pension diagnoses were available from 1994 and sick-leave diagnoses from 2005. Among identified MS patients, the distribution of costs between MS and other diagnosis groups, during 2010, was discerned using ICD-10 codes. Information regarding sociodemographics for each of the MS patients was obtained from the Longitudinal Integration Database for Health Insurance and Labor Market Studies (LISA) database at Statistics Sweden.

The Regional Ethical Review Board in Stockholm approved of the project (2007/762-31; 2014/236-32).

### Costs

The cost estimates were prevalence based, i.e., they included the costs during 2010, irrespective of whether the prevalent sickness absence or disability pension began before or continued after 2010. Costs for hospitalizations were assigned to the date of discharge to calculate the prevalence-based costs during 2010.

Direct costs included dispensed prescription drugs and specialized in- and outpatient healthcare use. Costs for dispensed prescribed medication were identified from the Swedish Prescribed Drug Register [14], administrated by the National Board of Health and Welfare. Healthcare costs (including costs for medication administered at hospitals to patients) were calculated from the Diagnosis Related Group (DRG) codes from PAR. DRG is a tool for grouping patients based on similar resource use, using information about diagnoses, procedures performed, age, sex, and status at discharge [15]. DRG codes were translated to costs using DRG weights published annually by the National Board of Health and Welfare, and the national average cost per 1.0 DRG (SEK 45,430 in 2010 [16]).

For the estimation from the societal perspective, costs for prescription drugs included both patient cost and the reimbursement paid by the county councils. For each individual, patient copayments for visits to physicians in specialized healthcare (SEK 300 per visit, according to the Swedish Association of Local Authorities and Regions) were added up to the national ceiling (SEK 900 [9]), assuming that the reimbursement period starts on 1 January. The daily inpatient fee was added (SEK 80 per day in hospital for all aged ≥18 years [9]) for each day in hospital. Estimated indirect costs in the overall COI estimate were the productivity losses, identified from sick-leave benefits and disability pension benefits registered in the LISA database. In the analyses of costs by diagnosis codes, the productivity loss due to net days on sick leave and disability pension, registered at the Social Insurance Agency, was used, since the LISA database does not include sick leave or disability pension diagnoses. The indirect costs were calculated by the human capital approach [17], using age-adjusted mean wage [18] and social security contributions made by employers [19].

Costs paid by the county councils (for healthcare and medication) and by the Social Insurance Agency (sick leave and disability pension benefits) were estimated, as part of the overall COI. The estimate included the reimbursed drug costs from the Swedish Prescribed Drug Register and the healthcare costs estimated from DRG weights (excluding patient copayments and the daily inpatient fee). Benefits paid by the Social Insurance Agency included costs for sick leave and disability pension,^1^ i.e., transfers. These benefits should not be viewed as a sub-component of the estimated indirect costs but estimate the costs paid by the third party payer for reimbursing lost wages.

### Analyses

Prevalence of MS in the total population 20–64 years of age in Sweden (5,496,770 individuals [20]) was calculated. Descriptive socio-demographic characteristics of the MS patients were reported regarding sex, age-groups, educational level, country of birth, type of living area (based on population density), geographic region, prescription drug use, healthcare use, sick leave, and disability pension. The overall societal COI, distributed between MS and other diagnostic groups, among MS patients, was calculated based on the main diagnosis, for healthcare resources used, and for sick leave and disability pension, respectively. To identify differences by sex in the distribution of costs, both the overall cost in each diagnosis group and the average cost by each sex was calculated. Accumulation of costs by components was described graphically to indicate the distribution of costs among MS patients. Individuals were sorted by age and by COI per person, respectively. The average direct, indirect, and overall MS-related costs are presented. Comparisons of costs, by individual characteristics and socioeconomic groups, were made using two-tailed *t*-tests with unequal variances or ANOVA (statistical significance: *p* < 0.05). Due to skewness of cost data, 95% confidence intervals were calculated using a bootstrap with 1000 iterations. The main results are also presented in euros (average annual exchange rate 2010: 1 euro = SEK 9.54).

## Results

Of the 14,077 identified MS patients of working age, most were women (71%), born in Sweden (89%), and aged >45 years (62%) (Table 1). The identified MS patients correspond to a MS prevalence of 0.26%, or 256 per 100,000 individuals of working age. The estimated total COI of these patients in 2010 was SEK 3950 million (approximately 414 million euros). Cost components in the overall COI, and the share of the overall COI that were paid for by the county councils and the Social Insurance Agency, respectively, are reported in Table 2. Indirect costs corresponded to 75% of the total COI.

**Table 1:** Description of the study population characteristics

	MS patients
	*n* (%)
Sex	
Men	4130 (29)
Women	9947 (71)
Age groups	
20–24 years old	280 (2)
25–34 years old	1691 (12)
35–44 years old	3387 (24)
45–54 years old	4217 (30)
55–64 years old	4502 (32)
Education	
≤9 years	1946 (14)
10–12 years	6873 (49)
≥13 years	5196 (37)
missing	*62 (0.4)*
Country of birth	
Sweden	12,580 (89)
Other than Sweden	1497 (11)
Type of living area*	
Larger cities	5189 (37)
Medium-sized municipalities	4927 (35)
Smaller municipalities	3961 (28)
Geographic region**	
East Sweden	5330 (38)
South Sweden	6054 (43)
North Sweden	2693 (19)
Healthcare resource use, during 2010***	
≥5 prescription drugs	7534 (54)
≥1 specialized outpatient visit	10,618 (75)
≥1 hospitalization	2992 (21)
Income, during 2010***	
Disposable income (mean±SD), SEK	185,854 ± 215,793****
Any sick leave	2861 (20)
Any disability pension	7263 (52)

*SD* standard deviation, *MS* multiple sclerosis, *n* number of people

* Based on population density according to the H-region classification scheme: larger cities (H1-H2), medium-sized municipalities (H3-H4), or smaller municipalities (H5-H6) [45]

** Based on Eurostat’s Nomenclature of Territorial Units for Statistics classification (NUTS1): East Sweden (SE1), South Sweden (SE2), or North Sweden (SE3) [46]

*** Figure does not add up to 100%, only one category reported

**** Excluding 33 individuals with negative disposable income and 41 individuals with zero values on disposable income. Disposable income is calculated for each individual from the family’s total income, including earnings, benefits, and other sources

**Table 2:** Overall COI from the perspective of the society, and costs paid by the counties and the Social Insurance Agency, for MS patients

	Overall societal COI	Of which paid by county councils and the Social Insurance Agency (i.e., transfers)
	SEK (%)	SEK (% of total*)
Prescription drug use	583,441,517 (15)	560,027,154 (96)**
Outpatient specialized healthcare use	140,758,388 (4)	133,977,488 (95)**
Inpatient healthcare use	271,472,848 (7)	269,125,488 (99)**
Direct costs	986,544,512 (25)	N/A
Sick leave***	328,809,660 (8)	137,240,200 (42)****
Disability pension	2,634,726,838 (67)	799,137,300 (30)****
Indirect costs	2,963,536,506 (75)	N/A
COI	3,950,081,018 (100)	N/A

The exchange rate is approximately SEK 10 to 1 euro

*COI* cost-of-illness, *MS* multiple sclerosis, *N/A* not applicable, *SEK* Swedish krona

* Proportion of the total cost for each cost category, compared to the overall COI

** Direct costs not accounted for are the patients’ copayments for prescription drugs and healthcare

*** Calculated based on all sickness benefits, i.e., sickness benefit, preventive sickness benefit, work injury benefit, and rehabilitation compensation

**** The transfer costs are not part of the indirect costs (which indicate the lost production to the employers) but an estimate of the payments made by the society/third part payer, in this case the Social Insurance Agency, to cover wages unpaid by employers due to sickness among their employees

Of all direct healthcare costs among the MS patients, 38% were for resource use with MS as the main diagnosis (Table 3). Based on main diagnosis groups, diseases of the nervous system (including MS), and of the genitourinary system represented diagnoses with the highest resource use in the MS patients, overall and both among women and men. Among men, mental disorders were also among the diagnoses with the highest resource use, while among women, ‘symptoms, signs and abnormal clinical and laboratory findings, not elsewhere classified’, had high resource use.

**Table 3:** The distribution of direct healthcare costs paid by the county councils and other authorities (excluding patient copayments) among MS patients, by main diagnosis group, based on diagnoses for specialized in- and outpatient healthcare use, registered in the National Patient Register from the National Board of Health and Welfare

ICD-10 chapters for diagnoses during specialized healthcare use	All MS patients (*n* = 14,077)	Men (*n* = 4130)	Women (*n* = 9947)
	Total cost SEK	Mean cost (95% CI) SEK	Mean cost (95% CI) SEK
01. Certain infectious and parasitic diseases (A00–B99)	1,087,9135	904 (662; 1146)	718 (425; 1011)
02. Neoplasms (C00–D48)	17,267,429	1072 (649; 1495)	1291 (870; 1712)
03. Diseases of the blood and blood-forming organs and certain disorders involving the immune mechanism (D50–D89)	1,153,613	27 (−2; 56)	105 (57; 153)*
04. Endocrine, nutritional and metabolic diseases (E00–E90)	5,299,977	287 (175; 398)	414 (320; 508)
05. Mental and behavioral disorders (F00–F99)	24,864,113	2942 (1140; 4743)	1278 (681; 1875)
06. Diseases of the nervous system (G00–G99)	172,203,772	13,095 (11,959; 14,231)	11,875 (11,144; 12,607)
Of which MS (G35)	152,551,326	11,715 (10,629; 12,800)	10,473 (9844; 11,101)
07. Diseases of the eye and adnexa (H00–H59)	5,328,371	380 (299; 460)	378 (321; 434)
08. Diseases of the ear and mastoid process (H60–H95)	1,014,547	53 (32; 73)	80 (59; 102)
09. Diseases of the circulatory system (I00–I99)	16,523,681	1743 (1136; 2349)*	938 (632; 1243)
10. Diseases of the respiratory system (J00–J99)	16,721,353	1584 (1138; 2029)	1024 (653; 1394)
11. Diseases of the digestive system (K00–K93)	16,372,195	1354 (1016; 1692)	1084 (808; 1359)
12. Diseases of the skin and subcutaneous tissue (L00–L99)	6,238,720	549 (322; 776)	399 (286; 512)
13. Diseases of the musculoskeletal system and connective tissue (M00–M99)	16,305,363	1231 (886; 1576)	1128 (942; 1314)
14. Diseases of the genitourinary system (N00–N99)	25,681,825	1800 (1444; 2155)	1835 (1563; 2106)
15. Pregnancy, childbirth and the puerperium (O00–O99)	9,568,775	0	962 (828; 1096)*
16. Certain conditions originating in the perinatal period (P00–P96)	5851	1 (−1;3)	0,2 (−0.2; 0.5)
17. Congenital malformations, deformations and chromosomal abnormalities (Q00–Q99)	483,225	48 (−18; 114)	29 (5; 53)
18. Symptoms, signs and abnormal clinical and laboratory findings, not elsewhere classified (R00–R99)	17,422,737	1053 (874; 1232)	1314 (1095; 1534)
19. Injury, poisoning and certain other consequences of external causes (S00–T98)	18,769,854	1489 (1151; 1826)	1269 (1072; 1466)
20. External causes of morbidity and mortality (V01–Y98)	0	0	0
21. Factors influencing health status and contact with health services (Z00–Z99)	18,774,568	1529 (805; 2253)	1253 (1035; 1470)
22. Codes for special purposes (U00–U99)	1890	0.5 (−0.4;1)	0
Missing information on main diagnosis	2,221,981	162 (92; 232)	156 (86; 226)
**Total cost**	**403,102,976**	31,300 (28,619; 33,981)*	2**7,529 (26,078; 28,981)**
Costs with MS as the main diagnosis	152,551,326	11,715 (10,629; 12,800)	10,473 (9844; 11,101)
Proportion with MS as the main diagnosis	38%	37%	38%

The exchange rate is approximately SEK 10 to 1 euro

*95% CI* 95% confidence interval, *ICD*-*10* International Classification of Disease version 10, *MS* multiple sclerosis, *SEK* Swedish Krona

* Statistically significant difference between groups (*p* < 0.05)

On the other hand, resource use with MS as the main diagnosis represented 67% of the indirect costs from sick leave and disability pension (Table 4). Mental disorders, diseases of the nervous system (including MS), and musculoskeletal disorders were diagnoses associated with high losses of productivity, overall, and among both women and men.

**Table 4:** The distribution of indirect costs among MS patients, by main diagnosis group based on diagnoses for sick leave or disability pension registered in the MiDAS register from the Social Insurance Agency

	All MS patients (*n* = 14,077)	Men (*n* = 4130)	Women (*n* = 9947)
ICD-10 chapters for sick leave and disability pension diagnoses	Total cost SEK	Mean cost (95% CI) SEK	Mean cost (95% CI) SEK
01. Certain infectious and parasitic diseases (A00–B99)	8,027,649	473 (40; 906)	611 (322; 899)
02. Neoplasms (C00–D48)	16,132,497	1295 (632; 1958)	1084 (711; 1457)
03. Diseases of the blood and blood-forming organs and certain disorders involving the immune mechanism (D50–D89)	1,644,059	0	165 (44; 286)*
04. Endocrine, nutritional and metabolic diseases (E00–E90)	11,678,481	976 (344; 1608)	769 (424; 1114)
05. Mental and behavioral disorders (F00–F99)	187,198,845	11,529 (9441; 13,617)	14,033 (12,585; 15,481)*
06. Diseases of the nervous system (G00–G99)	2,048,641,497	142,563 (136,733; 148,393)	146,764 (143,281; 150,246)
Of which multiple sclerosis (G35)	1,901,878,722	131,868 (126,171; 137,564)	136,450 (132,973;139,926)
07. Diseases of the eye and adnexa (H00–H59)	10,796,939	527 (115; 938)	867 (537; 1196)
08. Diseases of the ear and mastoid process (H60–H95)	7167,714	442 (83; 801)	537 (266; 808)
09. Diseases of the circulatory system (I00–I99)	33,285,976	2562 (1599; 3524)	2283 (1713; 2853)
10. Diseases of the respiratory system (J00–J99)	8,595,263	420 (57; 782)	690 (381; 998)
11. Diseases of the digestive system (K00–K93)	9,972,129	682 (194; 1171)	719 (388; 1050)
12. Diseases of the skin and subcutaneous tissue (L00–L99)	6,847,434	312 (-9; 632)	559 (267; 851)
13. Diseases of the musculoskeletal system and connective tissue (M00–M99)	185,300,465	7911 (6331; 9490)	15,344 (13,865; 16,824)*
14. Diseases of the genitourinary system (N00–N99)	3,260,885	137 (−76; 351)	271 (87; 454)
15. Pregnancy, childbirth and the puerperium (O00-O99)	5,018,137	0	459 (243; 665)*
16. Certain conditions originating in the perinatal period (P00-P96)	848,448	0	85 (−31; 202)
17. Congenital malformations, deformations and chromosomal abnormalities (Q00–Q99)	4,715,709	539 (93; 984)	296 (86; 506)
18. Symptoms, signs and abnormal clinical and laboratory findings, not elsewhere classified (R00–R99)	37,556,667	2406 (1449; 3363)	2777 (2163; 3391)
19. Injury, poisoning and certain other consequences of external causes (S00–T98)	47,106,270	3534 (2449; 4619)	3268 (2605; 3931)
20. External causes of morbidity and mortality (V01–Y98)	927,300	0	93 (−30; 217)
21. Factors influencing health status and contact with health services (Z00–Z99)	8,113,739	655 (196; 1113)	544 (308; 780)
22. Codes for special purposes (U00–U99)	0	0	0
Missing information on main diagnosis	182,624,342	12,011 (9778; 14,244)	13,373 (11,874; 14,871)
**Total cost**	**2,825,460,444**	**188,972 (183,122; 194,923)**	205,590 (201,886; 209,295)*
Costs with MS as the main diagnosis	1,901,878,722	131,868 (126,171; 137,564)	136,450 (132,973; 139,926)
Proportion with MS as the main diagnosis	67%	70%	66%

The exchange rate is approximately SEK 10 to 1 euro

*95% CI* 95% confidence interval, *ICD*-*10* International Classification of Disease version 10, *MS* multiple sclerosis, *SEK* Swedish krona

* Statistically significant difference between groups (*p* < 0.05)

**Fig. 1:**
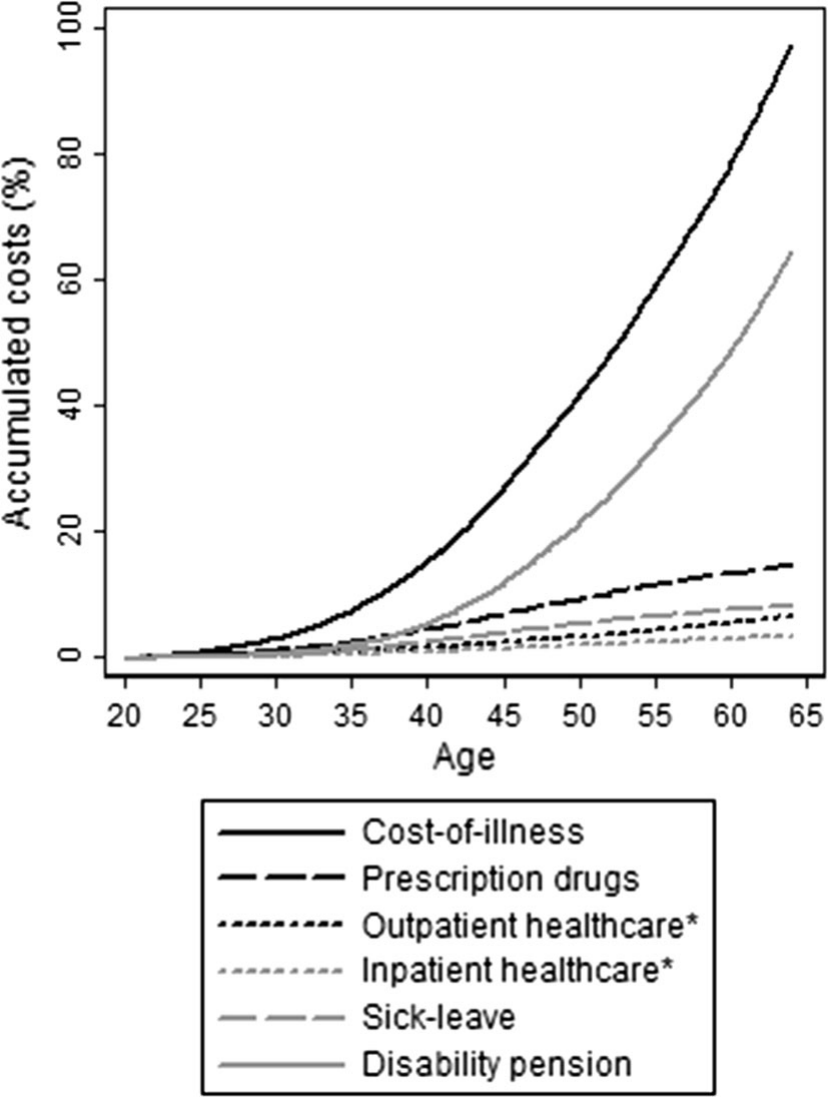
Distribution of the overall cost-of-illness of individuals with multiple sclerosis, accumulated by order of age. *Asterisk* healthcare costs in this figure do not include patient copayments, but are the costs paid by the counties and other authorities

**Fig. 2:**
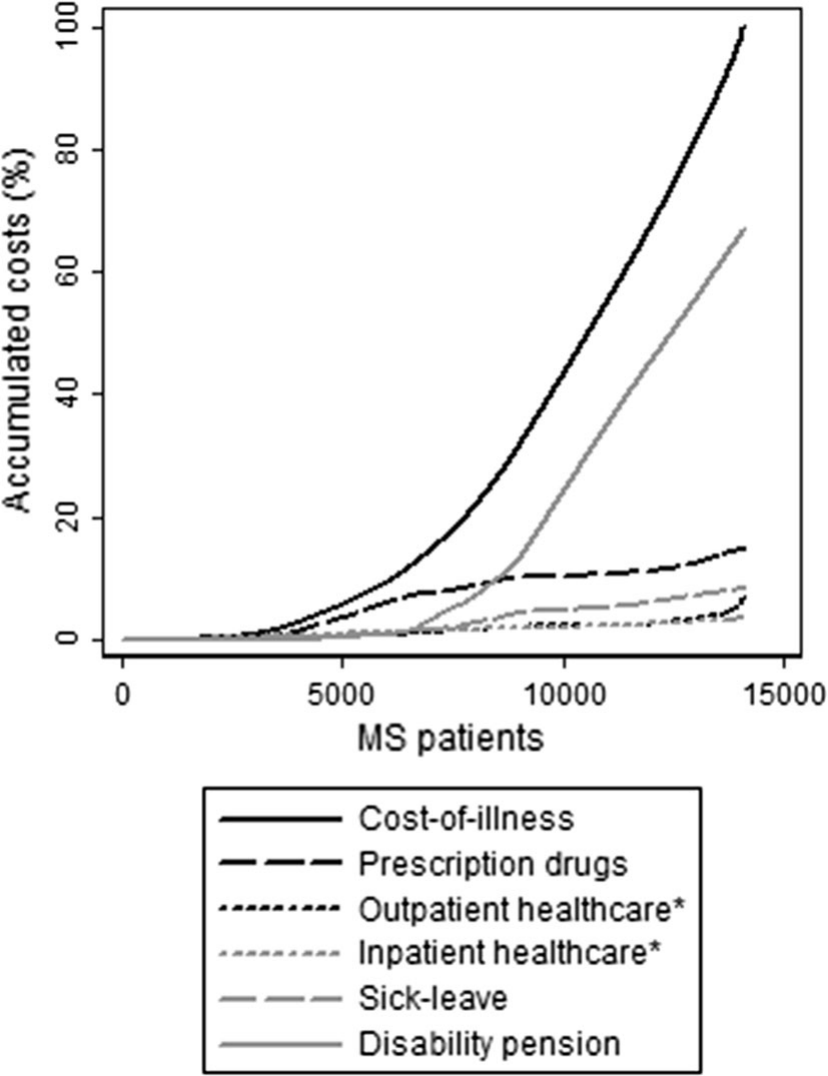
Distribution of the overall cost-of-illness of individuals with multiple sclerosis by order of accumulated overall cost-of-illness. *Asterisk* healthcare costs in this figure do not include patient copayments, but are the costs paid by the counties and other authorities

The distribution by age showed a higher accumulation of costs from disability pension among the older MS patients (Fig. 1). The distribution of costs was skewed among individuals with MS (Fig. 2 shows the distribution of costs for each cost component, after sorting MS patients from low to high COI), with 25% of the population (the 3509 persons to the far right in the figure) contributing to half the total COI. The accumulation of prescription drug costs appeared to occur among MS patients with lower COI.

Direct healthcare costs with MS as the main diagnosis were higher among young adults, those born outside Sweden, living in larger cities, or living in the south of Sweden, compared to other groups (Table 5). Indirect costs with MS as the main diagnosis were higher among those of older age, low education, born in Sweden, living in small municipalities, living in the north of Sweden, or having had MS for >8 years.

**Table 5:** Average overall, direct, and indirect costs with MS as the main diagnosis among MS patients, by individual characteristics and socioeconomic groups

	Overall costs*	Direct healthcare costs**	Indirect costs
	SEK (95% CI)	SEK (95% CI)	SEK (95% CI)
Sex	N.S.	N.S.	N.S.
Men	143,582 (137,655; 149,510)	11,715 (10,629; 12,800)	131,868 (126,171; 137,564)
Women	146,922 (143,314; 150,530)	10,473 (9844; 11,101)	136,450 (132,973; 139,926)
Age groups	(*p* < 0.001)	(*p* < 0.001)	(*p* < 0.001)
20–24 years old	31,731 (22,618; 40,843)	16,156 (11,332; 20,980)	15,575 (9397; 21,753)
25–34 years old	61,704 (56,159; 67,249)	16,098 (14,073; 18,123)	45,606 (40,719; 50,493)
35–44 years old	111,832 (106,214; 117,449)	13,113 (11,760; 14,467)	98,718 (93,411; 104,026)
45–54 years old	160,848 (155,085; 166,612)	10,232 (9296; 11,169)	150,616 (145,075; 156,157)
55–64 years old	196,387 (190,717; 202,057)	7384 (6638; 8130)	189,003 (183,486; 194,521)
Education	(*p* < 0.001)	N.S.	(*p* < 0.001)
≤9 years	182,424 (173,461; 191,386)	10,702 (9285;12,119)	171,722 (162,942;180,501)
10–12 years	162,481 (157,881; 167,081)	10,809 (10,037; 11,582)	151,672 (147,202; 156,141)
≥13 years	111,395 (106,957; 115,834)	10,948 (10,079; 11,817)	100,447 (96,186; 104,709)
Country of birth	(*p* = 0.012)	(*p* < 0.01)	(*p* < 0.001)
Sweden	147,325 (144,092; 150,557)	10,457 (9877; 11,038)	136,868 (133,737; 139,998)
Other than Sweden	134,325 (124,646; 144,004)	14,028 (11,980; 16,076)	120,298 (110,937; 129,658)
Type of living area	(*p* < 0.001)	(*p* < 0.001)	(*p* < 0.001)
Larger cities	129,100 (124,155; 134,046)	12,072 (11,041; 13,104)	117,028 (112,272; 121,783)
Medium-sized municipalities	150,827 (145,875; 155,779)	10,634 (9742; 11,527)	140,193 (135,366; 145,020)
Smaller municipalities	161,930 (155,862;167,998)	9470 (8614;10,327)	152,459 (146,535;158,384)
Geographic region	(*p* < 0.001)	(*p* < 0.001)	(*p* < 0.001)
East Sweden	135,612 (130,811; 140,412)	8718 (7909; 9526)	126,894 (122,275; 131,513)
South Sweden	149,688 (144,893;154,482)	14,129 (13,105;15,153)	135,558 (130,955;140,162)
North Sweden	157,969 (150,965; 164,974)	7630 (6828; 8432)	150,558 (143,427; 157,251)
Years since diagnosis	(*p* < 0.001)	N.S.	(*p* < 0.001)
0–7 years	80,197 (76,855; 83,539)	11,166 (10,408; 11,924)	69,031 (65,888; 72,175)
≥8 years	213,400 (208,637; 218,163)	10,499 (9691; 11,308)	202,901 (198,250; 207,551)
Average cost	145,942 (142,891; 148,994)	10,837 (10,283; 11,390)	135,105 (132,126; 138,085)

The exchange rate is approximately SEK 10 to 1 euro

*95% CI* 95% confidence interval, *COI* cost-of-illness, *MS* multiple sclerosis, *N.S.* not statistically significant, *SEK* Swedish krona

* Excluding prescription drug costs and patient copayments

** Excluding patient copayments

## Discussion

This large and population-based study demonstrates that the overall COI of all the approximately 14,000 individuals of working age with MS in 2010 was estimated as SEK 3950 million, or SEK 281,000 per MS patient, of which 75% consisted of indirect costs. The total COI corresponds to more than 1% of the total annual expenditure on health care in Sweden.^2^ Direct costs were to a large extent covered by reimbursements from the county councils, while sickness benefits did not give a comprehensive picture of the impact of MS on lost productivity in Sweden. More than half of the COI was for resource use with MS as the main diagnosis. Our mapping of diagnosis groups shows that in addition to nervous system disorders (including MS), mental and musculoskeletal disorders contributed largely to the resource use in this group of patients. The distribution of costs included in the COI estimate was skewed in the population with MS, and different segments of the population with MS contributed to different types of resource use.

The strengths of this explorative, prevalence-based study included the use of data from several nationwide registers of high quality [21, 22], which captured a large number of MS patients and allowed for subgroup analyses. The calculated MS prevalence (256 per 100,000 individuals) was high compared to previous estimates from Europe (56–232 per 100,000 individuals [1] and 189 per 100,000 in Sweden [23]), although the estimated number of individuals with MS in Sweden is comparably low (14,077 in our study, compared to 17,500 individuals of all ages with MS in Sweden [23]). This is probably due to the fact that the focus of this study is people of working age, thus excluding children/adolescents who have a lower prevalence [23], and older MS patients included in previous estimates.

Limitations are that the data did not include information regarding healthcare use delivered by primary healthcare or municipalities, nor were patient copayments for healthcare use included in the registers, but were estimated from average fees for healthcare encounters and based on an assumption about the reimbursement period and ceiling of healthcare copayments. Although this assumption neglects the copayments in primary care and thus overestimates the copayments, specialized outpatient care represents a large proportion of outpatient care in this patient group [24] and copayments are higher per visit in specialized outpatient care compared to primary care. Regarding methods used for calculation of costs, the DRG weights and the national average cost per DRG were used as proxies for healthcare costs. Thus, our results give an indication of the expected costs associated with healthcare among individuals with MS.

Costs for drugs administered within healthcare clinics (i.e., indented drugs) are also not included, because these are not available in the Swedish Prescribed Drug Register (but may to some extent be covered by DRGs). This concerns many of the injection drugs used for MS and will thus result in an underestimation of the COI among MS patients. Data from the Swedish Multiple Sclerosis register (SMSreg) indicate that in 2010, some 1100 patients were continuously on natalizumab (an infusion drug used exclusively for MS and administered in healthcare units; not prescribed to patients). With a price of approximately 200,000 SEK per year for natalizumab, this means that the estimated societal cost for MS drugs was underestimated by SEK 200 million (approximately 5% of the estimated COI).

In this study, sick leave paid by the employer (usually the first 14 days of a sick-leave spell) and not reimbursed by the Social Insurance Agency, was not included. Thus, the indirect costs were slightly underestimated. Half of the MS patients of working age had disability pensions and 20% had (reimbursed) sickness absence at least once during 2010, which was in line with previous results from Ireland, where 25% of MS patients worked full-time and 20% had no financial support due to the disease [25]. However, it is the long sick-leave spells that generate the highest costs. Concerning indirect costs, it has been suggested that the human capital method for calculating indirect costs overestimates the productivity losses from disease [26]. The alternative methods have, however, also been criticized [27]. Based on our calculated cost paid by the Social Insurance Agency for sick leave and disability pension, a more conservative estimate is provided, although this does not correspond to lost productivity but to actual transfer payments to patients on sick leave and disability pension.

Moreover, costs not available in nationwide registers should also be accounted for, as part of the economic impact of MS, including long-term care, early mortality costs, and intangible costs resulting from MS [28]. Previous studies of bottom-up design, using patient inquiries, have identified resource use for both formal and informal home care [29], and intangible costs associated with MS relapses [30], which were not available in the current study. It has been reported that approximately 20% of patients changed residence due to MS and that 35% required assistance of more than 1 h daily [25]. According to previous studies, social services and intangible costs have been suggested to represent 20–50% of the overall COI of MS [30]. In particular, costs for community services, such as personal assistants, appears to represent a large cost component among Swedish MS patients in more severe disease states [5].

Thus, our results of COI of MS are probably an underestimation, as some resource use was not included, which needs to be taken into account when interpreting and comparing results between studies. This study presents an attempt at identifying costs for all patients with MS on a national level, using national registers of costs, a novel method that has previously only been reported for Danish MS patients [31]. Future studies of the full economic impact of MS are thus warranted (and possible), combining data from *top*-*down studies* based on nationwide registers and *bottom*-*up studies* using comprehensive data on resource use collected from patients through, e.g., questionnaires. These are two different approaches for COI studies [32], and according to a recent literature review the currently available knowledge of costs among MS patients is almost entirely based on data from patient surveys with unclear representativeness in the total population of MS patients [4]. It has previously been suggested that survey studies among MS patients have been biased towards patients with moderate or severe disease and may thus not fairly represent resource use among patients with less severe MS disease [3, 33].

Most studies in COI of MS cover other age groups, e.g., include also older patients, or include also other types of costs, e.g., primary healthcare or home care. As the bulk of indirect costs (sick leave and disability pension) only concern people of working age, it is important to study COI especially for this age group. When comparing our results to those of other studies using a top-down methodology (i.e., based on data from nationwide registers), our result (SEK 3950 million/ 414 million euros) is similar to the estimated costs of MS for all ages in Sweden in 1994: SEK 1736 million [24] (equals approximately SEK 3100 million in 2010 values using the Swedish healthcare inflation index). In contrast, our results (≈29,400 euros/patient) are fairly high compared to the estimated costs in Denmark: 14,575 euros per MS patient in 2006 [31]. However, that study included other resource use as well as MS patients of all ages, thus making comparisons of average indirect costs between the studies unfeasible. In all, our estimated proportion of indirect costs in the COI (75%) was comparable to previous estimates from top-down studies of MS: 80% in Sweden [24], and 76% in Denmark [31]. In contrast, the most recent bottom-up study (mainly based on patient inquiries) for MS in Sweden identified 68% direct costs [3], of which a large proportion were costs for personal assistance. In all, our estimates are much lower compared to studies using bottom-up methods, resulting in annual costs of MS for all ages in Sweden of 586 million euros for 1998 [34], and 600 million euros in 2005 values [3]. However, those studies also included primary healthcare visits, informal care, social services, investments/adaptations, and short-term absence [3, 34]. Thus, the resulting difference should in part be the difference in cost components included, and in part be the result of how costs are identified [4, 7], which indicates the methods are complementary.

Our annual COI estimate is the full cost of the included cost components for MS patients, while most bottom-up studies seek to identify the costs resulting only from MS. However, we also reported the costs resulting from resource use with MS as the main diagnosis. Our estimated annual COI should, thus, be an overestimation of the COI of MS, as it does not exclude costs for other diagnosis groups, while the presented costs resulting from MS in this study will be an underestimation as it does not take into account, e.g., drug costs and resource use for which MS was a secondary diagnosis. This is an important caution, as a disease like MS, with known prevalent comorbidities [35], may result both in increasing resource use during encounters for other diseases of differing etiology, and be an underlying cause of other diseases and diagnoses. Moreover, resource use during encounters with MS as the main diagnosis may be increased by ongoing comorbidities [36]. Future analyses of how comorbidities among MS patients affect the resource use for each condition are warranted. Estimating the full COI enabled us to distinguish additional costs resulting from resource use with other main diagnoses among MS patients, but the results need to be interpreted with the issues related to main diagnoses and secondary diagnoses in mind.

Thus, we found that mental disorders, diseases of the nervous system (also when excluding MS), musculoskeletal disorders, and genitourinary disorders, were the diagnosis groups associated with the highest costs among MS patients in Sweden. MS has been associated with higher rates of, e.g., bipolar disorder and depression (both included in the group mental disorders) [37]. Moreover, gastrointestinal, musculoskeletal, ocular, pulmonary, and renal disorders are common among MS patients [38], of which, in particular, gastrointestinal, pulmonary and renal disorders contributed to the overall COI in our study. When MS patients in Sweden were older compared to the general population of working age (e.g., 62% above 45 years of age vs 44% in the general population of working age [20]), and more often had at least five prescribed drugs (54 vs 8% [39]), comorbidities were to be expected. Moreover, it has been reported that comorbidities, in particular hypertension, diabetes, ischemic heart disease, chronic lung disease, depression, and bipolar disorder, increase the hospitalization rate (for all causes) among MS patients [36], which may contribute to the overall high COI among individuals with MS. This can also be seen in the high number of MS patients with sick leave or disability pension (20 and 54% vs 8 and 8%, respectively, in the general population of working age [40]), and the low average income of MS patients, below the median of the general population of working age (SEK 227,400 [20]). More research is warranted on how the combined effect of MS and comorbidities among MS patients impacts COI in these patients, including different types of comorbidities and associations between MS and other diseases.

The overall COI was skewed among individuals with MS: costs for, e.g., hospitalizations were more pronounced among those with high overall COI, and the large indirect costs resulting from disability pension were accumulated among the older in the study population. This is in line with previous findings of higher resource use among MS patients with higher disease severity [41], additional disease symptoms (such as spasticity [42]), and comorbidities, such as mental disorders or pain [12]. Probably, the individuals with high COI are more likely to have high severity of the disease. However, that cannot be identified from the registers available for our study.

Moreover, it appears that the high costs for drugs occur in a group of MS patients with fairly low COI but not necessarily of low age. This is in line with results from a systematic review [4], reporting that drugs contributed to a large proportion of the overall costs in MS patients with low severity of disease. According to our study it appears that individuals with high overall resource use, and thus probably high disease severity, did not contribute largely to the overall drug costs. Moreover, although the overall COI was higher among the older MS patients, the direct healthcare costs resulting from MS were highest among the younger patients in this study. This may be an indication that younger MS patients with recent disease onset are receiving disease modifying treatments, thus potentially resulting in high costs for healthcare encounters, including drug treatment, which is in line with the current Swedish and international treatment guidelines to use disease modifying treatment primarily in young MS patients with more active inflammation [43]. However, our data did not enable analyses about drug use during healthcare encounters. Future studies are warranted on the association between MS treatments and severity of disease and on the economic impact of MS. Moreover, the aim of this study was to identify the prevalence-based costs [44]. That is, other studies are needed for analyses of long-term costs over the MS trajectory.

## Conclusions

Based on register data, the indirect costs contribute to three fourths of the overall COI of MS patients of working age in Sweden. Counties and other authorities pay a large part of the direct costs. Although many MS patients also have other diagnoses, MS is the main diagnosis for healthcare and lost productivity resulting in more than 50% of the estimated costs. The different patterns in the distribution of direct and indirect costs warrant further studies to identify potential inequalities by socioeconomic factors among MS patients. However, the younger individuals’ higher direct costs may suggest that healthcare resources are particularly allocated to MS patients early during their disease, a period when MS-specific interventions are known to be most effective.

## Supplementary information

The authors of this study are not allowed to make the micro-level data in this study publically available due to its sensitive nature. According to the Swedish Ethical Review Act, the Personal Data Act, and the Administrative Procedure Act, data can be made available after legal review for researchers who meet the criteria for access to this type of sensitive and confidential data. For questions about this, please contact Professor Kristina Alexanderson, responsible for the data set.

## Footnotes

1 Sick-leave benefits account for 80% and disability pension for 64% of lost income, up to a certain level.

2 According to the System of Health Accounts, provided by Statistics Sweden, the total cost in 2010 was SEK 298,721 million, including somatic, psychiatric and dental curative and rehabilitative care, long-term care, medical goods such as prescribed drugs, preventive care, and governance and administration.
